# Insights on the Role of Putative Muscle-Derived Factors on Pancreatic Beta Cell Function

**DOI:** 10.3389/fphys.2019.01024

**Published:** 2019-08-08

**Authors:** Maria L. Mizgier, Rodrigo Fernández-Verdejo, Julien Cherfan, Michel Pinget, Karim Bouzakri, Jose E. Galgani

**Affiliations:** ^1^UMR DIATHEC, EA 7294, Centre Européen d’Etude du Diabète, Université de Strasbourg, Strasbourg, France; ^2^Departamento de Ciencias de la Salud, Nutrición y Dietética, Facultad de Medicina, Pontificia Universidad Católica de Chile, Santiago, Chile; ^3^Departamento de Nutrición, Diabetes y Metabolismo, Facultad de Medicina, Pontificia Universidad Católica de Chile, Santiago, Chile

**Keywords:** myokines, miRNA, exosomes, insulin secretion, insulin sensitivity, muscle, beta cell, crosstalk

## Abstract

Skeletal muscle is a main target of insulin action that plays a pivotal role in postprandial glucose disposal. Importantly, skeletal muscle insulin sensitivity relates inversely with pancreatic insulin secretion, which prompted the hypothesis of the existence of a skeletal muscle-pancreas crosstalk mediated through an endocrine factor. The observation that changes in skeletal muscle glucose metabolism are accompanied by altered insulin secretion supports this hypothesis. Meanwhile, a muscle-derived circulating factor affecting *in vivo* insulin secretion remains elusive. This factor may correspond to peptides/proteins (so called myokines), exosomes and their cargo, and metabolites. We hereby review the most remarkable evidence encouraging the possibility of such inter-organ communication, with special focus on muscle-derived factors that may potentially mediate such skeletal muscle-pancreas crosstalk.

## Introduction

Skeletal muscle is the largest organ in lean humans that plays a main physiological role in insulin-stimulated glucose disposal (Baron et al., 1988). To accomplish this, muscle cells adapt their metabolism to changes in circulating metabolites and hormones. Muscle cell adaptation also involves autocrine and paracrine interaction among cell types (Pillon et al., 2013). Thus, several muscle cell-derived factors have been detected, including myokines (Bouzakri et al., 2011; Raschke et al., 2013; Scheler et al., 2013; Hartwig et al., 2014; Mizgier et al., 2017), exosomes and their cargo (Forterre et al., 2014), and metabolites (Daskalaki et al., 2018). Whether or not muscle-derived factors increase in blood at a sufficient magnitude to influence distant organs has not yet been elucidated.

Among muscle-targeted organs, a crosstalk between skeletal muscle and the pancreas has been proposed (Bouzakri et al., 2011; Mizgier et al., 2017; Rutti et al., 2018). Pancreatic beta cells secrete insulin to facilitate glucose disposal in insulin-sensitive tissues such as skeletal muscle, so hyper- and hypo-glycemia are prevented. The extent at which insulin is secreted depends of the amount of glucose taken up by beta cells (Rorsman and Braun, 2013). Furthermore, beta cells receive information from other organs to adjust insulin secretion. For instance, when glycemia is low, the brain increases sympathetic activity to suppress insulin secretion, which raises glycemia back to normal levels (Porte, 1969).

 Whether a similar mechanism is activated under conditions of high glycemia (e.g., after meals) is unknown. Since skeletal muscle is the main tissue responsible for insulin-mediated glucose disposal (Baron et al., 1988), it is intuitive to propose that skeletal muscle can signal back toward the pancreas. Such organ crosstalk may allow adjusting insulin secretion to insulin demands to maintain glycemia within the physiological range. Here, we will discuss the most remarkable evidence supporting such a muscle-pancreas crosstalk, and the progress made in identifying the endocrine mediator.

## Physiological Relevance of A Putative Skeletal Muscle-Pancreas Crosstalk

Pancreatic insulin secretion must tightly match insulin demands for proper glycemic control. Upon glucose ingestion, the increase in glycemia and glucose transport into pancreatic beta cells may suffice to adapt insulin secretion to insulin demand. In turn, higher glycemia often observed in insulin resistance, especially in postprandial conditions (Petersen et al., 2004; Galgani and Ravussin, 2012), may also be sufficient to elevate insulin secretion. Thus, blood glucose concentration might itself be the factor contributing to adjust insulin secretion.

However, convincing evidence shows that blood glucose concentration cannot entirely accommodate insulin secretion to insulin demand (Kahn et al., 1989, 1990). Another physiological mechanism must therefore exist to maintain glycemic control. The skeletal muscle-pancreas crosstalk appears as an attractive hypothesis. The fact that insulin is secreted as an inverse function of insulin sensitivity under fasting and postprandial conditions (Galgani et al., 2014) reinforces the notion of such organ crosstalk (Mizgier et al., 2014). The mechanism underlying this association remains elusive. But, it may be mediated by a factor coming from skeletal muscle (and eventually from other insulin-sensitive tissues). Thus, skeletal muscle may play a pivotal physiological role in the adjustment of insulin secretion to maintain glycemic control throughout the day.

## Evidence Suggesting A Skeletal Muscle-Pancreas Crosstalk

A large-scale, multi-center study investigated which factors determine the change in insulin secretion (by plasma C-peptide concentration) during an isoglycemic-hyperinsulinemic clamp (Mari et al., 2011). The analysis highlighted insulin-stimulated glucose disposal rate as a direct determinant of insulin secretion. That finding was independent of sex, age, family history of diabetes and body mass index. Noteworthy, that association was attenuated in individuals with altered glycemic control. One can suggest that the extent to which glucose is taken up in skeletal muscle, as a main insulin-sensitive glucose disposal site (Baron et al., 1988), somehow determines pancreatic beta cell function. Alternatively, skeletal muscle insulin sensitivity may just reflect beta cell insulin sensitivity. The observation that insulinemia during the isoglycemic clamp related positively with insulin secretion suggests a direct action of insulin on its secretion (Mari et al., 2011). Pursuing that latter hypothesis, i.e., that insulin enhances its secretion through a direct effect on beta cell function, a sophisticated study was conducted in humans (Halperin et al., 2012).

The study compared the effect of saline vs. insulin infusion on glucose-stimulated insulin secretion (GSIS) in individuals with normal and abnormal glycemic control (Halperin et al., 2012). Blood glucose concentration was maintained at similar values in both sessions by co-infusing glucose throughout the insulin infusion session. When compared with saline, the insulin infusion increased GSIS in healthy individuals. Such effect was attenuated in volunteers with altered glycemic control. These results were interpreted as proof that insulin stimulates its own secretion in pancreatic beta cells. Furthermore, lower GSIS in subjects with altered glycemic control may fit with a state of beta cell insulin resistance. Taken together, these findings were considered in agreement with some *in vitro* data showing that insulin stimulates its secretion (Aspinwall et al., 1999; Srivastava and Goren, 2003; Jimenez-Feltstrom et al., 2004; Caporarello et al., 2017; Supplementary Table 1).

However, other studies have not confirmed that insulin (*in vitro*) stimulates its secretion (reviewed in Leibiger et al., 2008 and updated in Supplementary Table 1). For instance, a neutral effect was reported in isolated rat islets (Zawalich and Zawalich, 2002) and a rat beta cell line (INS-1) (Collier et al., 2004). Even more, an inhibitory action was concluded from experiments in perfused canine pancreases (Iversen and Miles, 1971); human islets (Johnson and Misler, 2002; Persaud et al., 2002); rat islets (Araujo et al., 2002); mouse beta cells; and INS-1 cells (Collier et al., 2004). Unpublished findings from our group also support an inhibitory action. Briefly, isolated mouse islets in which insulin signaling was inhibited (with wortmannin) displayed higher GSIS. This suggests that insulin secreted upon glucose stimulation decreased further insulin secretion.

Aforementioned findings *in vitro* are controversial (Supplementary Table 1), although it predominates an inhibitory action of insulin on its secretion. A study that assessed C-peptide release at different insulin concentrations in mouse islets might enlighten an answer. This study showed that insulin stimulated its secretion when tested at concentrations between 0.05 to 0.1 nM. In turn, insulin inhibited its secretion at a higher concentration (1 μM) (Jimenez-Feltstrom et al., 2004). Whether this dose-dependent effect of insulin on its secretion explains such controversial findings is elusive.

**FIGURE 1:**
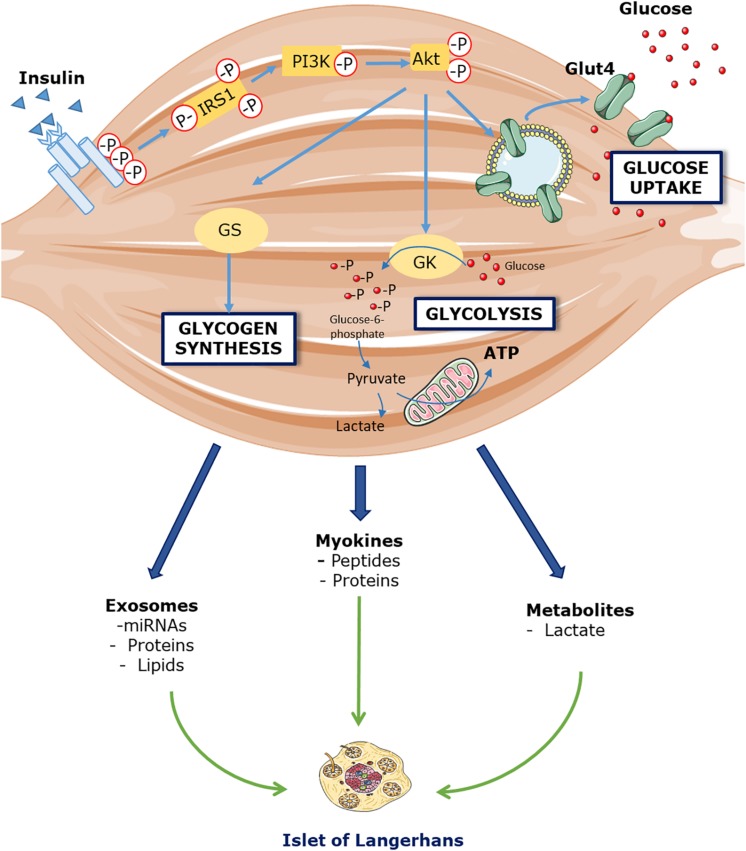
Hypothetical model linking skeletal muscle insulin sensitivity and pancreatic insulin secretion. Insulin binds to its receptor located in skeletal muscle cells that triggers phosphorylation of Akt, a pivotal protein involved in insulin signaling. As a consequence, GLUT4 are translocated from cytosol to cell membrane, which increases facilitated transport of glucose into muscle cells. Increased cellular glucose flux will enhance its disposal by increasing glycogen synthesis and glucose utilization for ATP synthesis. Changes in glucose disposal at the level of uptake, storage or oxidation may be the signal to trigger the release of a putative factor into circulation, which can then influence insulin secretion directly through its action on beta cells, or indirectly through extra-pancreatic cell types.

Now, if insulin indeed displays an inhibitory action on its own secretion *in vivo*, how can the increase in GSIS after insulin infusion be explained? (Halperin et al., 2012). From studies conducted by the same research group (Bouche et al., 2010; Lopez et al., 2011; Halperin et al., 2012), it is acknowledged that glucagon, cortisol, free-fatty acids or potassium are likely not responsible. We then propose that an endocrine factor signaling from skeletal muscle toward the pancreas may enhance insulin secretion. If insulin plays an inhibitory action on its secretion *in vivo*, this endocrine factor must be potent enough to overcome that inhibition. We speculate that the release into circulation of that factor is triggered by insulin itself or the increase in glucose disposal (Figure 1).

Two mouse models with conditioned expression of specific proteins in skeletal muscle support this muscle-pancreas crosstalk. A loss-of-function of peroxisome proliferator-activated receptor γ coactivator 1α (PGC1α) (Handschin et al., 2007) and a gain-of-function of muscle-specific RING-finger 1 protein (MuRF1) (Hirner et al., 2008) are accompanied by altered skeletal muscle glucose metabolism as well as abnormal insulin secretion *in vivo*. In the former study, the loss of PGC1α expression at the muscle level induced glucose intolerance in mice due to hypoinsulinemia. Such defect in *in vivo* insulin secretion was not detected in isolated islets, suggesting that a circulating muscle-derived factor present *in vivo* plays a role. In this case, IL-6 was suggested as a candidate. The second study showed that overexpression of MuRF1 was accompanied of lower hepatic glycogen content and hyperinsulinemia. The authors concluded that an alteration of MuRF1 expression in skeletal muscle stimulates insulin secretion, providing a regulatory feedback loop between muscle and pancreas. Besides, we found that conditioned medium from human myotubes increases insulin secretion in isolated pancreatic islets (Bouzakri et al., 2011; Mizgier et al., 2017). Moreover, the contribution of skeletal muscle on GSIS seems to be fiber specific (Rutti et al., 2018), adding further complexity to the muscle-pancreas axis. These data suggest that insulin secretion is influenced through muscle-derived factors released in response to modifications in skeletal muscle glucose metabolism. A muscle-to-pancreas communication therefore seems to exist.

## Muscle-Released Factors Targeting Insulin Secretion

### Myokines

Myokines having influence on insulin secretion should express their receptors in pancreatic islets. Because dozens and even hundreds of proteins have been detected in conditioned media from human muscle cells (Hartwig et al., 2014; Weigert et al., 2014), we first compared the presence of selected proteins in different studies (see Supplementary Table 2 for further details). Nineteen out of 73 (26%) proteins were consistently detected regardless of the detection method (Raschke et al., 2013; Scheler et al., 2013; Hartwig et al., 2014). Among secreted proteins, interleukins (IL) 1-beta, 2, 6, 7, 8, 10, and 12 stand out. Also, chemokines such as monocyte chemoattractant protein-1 (MCP1/CCL2); regulated on activation, normal T cell expressed and secreted (Rantes); and growth-regulated oncogene (GRO) alpha, beta and gamma. The proteins detected by at least two analytical approaches were selected for a gene expression search of their receptors in human, mouse and rat pancreatic islets and beta cells in the Beta Cell Gene Atlas (Smink et al., 2005; Table 1). Many of the selected proteins do express their receptors in islets or beta cells, which opens the number of potential endocrine candidates. Most of these proteins have often more than one receptor belonging to CC or CXC receptor families, which adds further complexity to the quest for endocrine muscle-pancreas mediators. As an example of the complexity, CXCL10 appears to interact with the toll-like receptor 4 but not with its known receptor (CXCR3) in beta cells (Schulthess et al., 2009). Additionally, myokine receptors may also be located in extra-pancreatic tissues. For instance, IL-6 binds to receptors located in intestinal L cells to stimulate glucagon-like peptide 1 secretion, which then can enhance GSIS (Ellingsgaard et al., 2011).

**TABLE 1:** Receptor expression in human, mouse and rat pancreatic islets and beta cells of cytokines and chemokines detected in conditioned media from human myotubes.

**Protein**	**Gene**	**Uniprot identifier**	**Receptor**	**mRNA expression**					
				
				**Beta cells**			**Islets**		
					
				**Human**	**Mouse**	**Rat**	**Human**	**Mouse**	**Rat**
GRO α/β/γ	CXCL1/2/3	P09341/P19875/P19876	CCR-1	−	−	−	−	++	−
			CCR-5	−	−	+	+	++	−
			CXCR-1	++	−	No data	No data	−	+
			CXCR-2	++	−	+	−	−	−
			CXCR-3	+++	−	++	++	+	+
IFN-γ	IFN-G	P01579	INFGR-1	++	+++	+++	++	+++	++
			INFGR-2	++	+++	+	++	+++	++
IL-1β	IL-1B	P01584	IL1R-1	+++	++	+++	+++	+++	+
			IL1R-2	+	+++	++	++	++	++
IL-2	IL-2	P60568	IL2R-α	−	−	+	+	+	−
			IL2R-β	−	+	−	++	++	+
IL-6	IL-6	P05231	IL6R	+	++	+++	+++	−	++
IL-7	IL-7	P13232	IL2R-γ	++	−	+++	−	++	+
			IL7R	++	No data	++	+	+	No data
IL-8	CXCL8	P10145	CXCR-1	++	−	No data	No data	−	+
			CXCR-2	++	−	+	−	−	−
			DARC	++	No data	++	++	−	No data
IL-10	IL-10	P02778	IL10R-α	++	+	−	−	++	−
			IL10R-β	+++	++	+++	++	+++	No data
IL-12	IL-12A/B	P29459/P29460	IL12R-β1	−	No data	+	−	+	No data
			IL12R-β2	+	+	++	++	+	No data
LIF	LIF	P15018	LIFR	−	+	+	++	++	+
M-CSF	CSF1	P09603	CSF1R	+	+	+++	+	++	+
MCP-1	CCL2	P13500	CCR-1	−	−	−	−	++	−
			CCR-2	No data	−	++	+	No data	−
			CCR-5	−	−	+	+	++	−
MIF	MIF	P14174	CXCR-2	++	−	+	−	−	−
			EGFR	+	+++	+++	++	+++	+++
RANTES	CCL5	P13501	CCR-1	−	−	−	−	++	−
			CCR-2	No data	−	++	+	No data	−
			CCR-3	+	+	−	−	−	+
			CCR-4	−	+	+	−	−	+
			CCR-5	−	−	+	+	++	−
			GPR75	No data	+	−	+	+	No data
VEGF-A	VEGFA	P15692	VEGFR-1	++	No data	+++	+	+++	No data
			VEGFR-2	+	++	+++	+	++	++
G-CSF		P09919	CSF3R	+	+	No data	+	++	No data
IL-13		P35225	IL13RA1	+	++	++	+++	++	+++
TNF-α		P01375	TNFRSF1A/TNFR1	+++	+++	+++	+++	+++	+++
			TNFRSF1B/TNFBR	+++	+	−	+	++	−
IL-4		P05112	IL4R	++	+	−	+++	+	−
			IL13RA1	+	++	++	+++	++	+++

*Specific receptor name for each cytokine and chemokine detected in human myotube conditioned media in Hartwig et al. (2014) and Mizgier et al. (2017) studies (see Supplementary Table 2) were identified browsing the UniProt and STRING protein databases. Receptor mRNA expression were obtained from the Beta Cell Gene Atlas (Smink et al., 2005). Low (+), moderate (++), enriched (+++) expression level; not expressed (−).*

The effect of specific myokines on beta cell function has been assessed by using their recombinant forms. IL-6 showed a stimulatory action on GSIS in mice after an acute injection (400 ng) that emulated its circulating concentration in response to exercise or high-fat diet (Ellingsgaard et al., 2011). By contrast, neutral (Ellingsgaard et al., 2008) or inhibitory (Handschin et al., 2007) actions are also reported. These controversial outcomes might be due to different IL-6 levels achieved. Physiological concentrations of IL-6 (<100 pg/mL) had anti-inflammatory effect and show a stimulatory action on GSIS (Andreozzi et al., 2006; Ellingsgaard et al., 2011). In turn, higher IL-6 concentrations (500–25000 pg/mL) show inhibitory action on GSIS (Sandler et al., 1990; Southern et al., 1990; Eizirik et al., 1994).

Additional cytokine assessment includes IL-12, CXCL10 and fractalkine (CX3CL1). Both IL-12 and CXCL10 have shown to impair GSIS in INS-1 cells (Taylor-Fishwick et al., 2013) and in human islets (Schulthess et al., 2009; Taylor-Fishwick et al., 2013). In turn, fractalkine at 100 ng/mL increased GSIS in human islets and in a mouse beta cell line (MIN6) (Lee et al., 2013). However, fractalkine did not influence GSIS when added at a lower concentration in sorted rat beta cells or human islets (Rutti et al., 2014), although it prevented tumor necrosis factor alpha-induced GSIS reduction in sorted rat beta cells (Rutti et al., 2014). The type of receptor also plays a role in determining the action of its ligand. For instance, Rantes appears to have a stimulatory effect when bound to GPR75 receptor (Liu et al., 2013), while an inhibitory action when bound to CCR1 receptor (Pais et al., 2014).

An additional aspect to take into account when searching for potential endocrine mediators is to distinguish among proteins released from cell lysis vs. an active, regulated secretion process. In fact, many detected proteins do not seem to be secreted as they have no signal peptide, known receptor and their described function is at the intracellular level (Supplementary Table 3). Thus, the myokinome may include a much lower number of proteins than previously anticipated.

### Exosomes and Their Cargo

Extracellular vesicles are a heterogeneous population of cell-derived membranous structures including microvesicles and exosomes. Exosomes are the smallest vesicles (ranging from 30 to 100 nm of diameter) formed by the fusion of multivesicular endosomes with the plasma membrane (van Niel et al., 2018). Exosomes carry proteins, lipids and nucleic acids [e.g., micro (mi)RNAs] (van Niel et al., 2018). Released exosomes can dock to the plasma membrane of a target cell, where they can fuse with the plasma membrane or be endocytosed before delivering its cargo (Raposo and Stoorvogel, 2013). Some evidence suggests a role in cell-to-cell and organ-to-organ communication, particularly through miRNAs (Camussi et al., 2010). However, the role of exosomes in whole-body homeostasis remains unclear.

Skeletal muscle cells can release exosomes (Forterre et al., 2014) and miRNAs (contained or not in exosomes) (Aoi and Sakuma, 2014). Furthermore, exercise and metabolic diseases such as type 2 diabetes (T2D) affect the expression of several muscle-derived miRNAs (myomiRs). For instance, miR-1 and miR-133a expressions are increased by acute endurance exercise in untrained subjects while miR-133b and miR-206 are not affected by acute exercise (Nielsen et al., 2010). In turn, all of these myomiRs were downregulated after a 12 week training period (Nielsen et al., 2010). Other myomiRs such as miR-23b/27b are down-regulated during muscle cell differentiation in individuals with T2D vs. healthy subjects (Henriksen et al., 2017), while the miR-29 family is up-regulated in muscle from T2D vs. non-diabetic donors (Massart et al., 2017).

Besides myomiRs expression being affected by exercise or diabetic status, exosome-associated miRNAs can also be transferred to other cell types. In this regard, Jalabert et al. (2016) found that skeletal muscle-derived exosomes injected into mice specifically targeted pancreatic islet cells and affected gene expression and proliferation of beta cells. Whether or not skeletal muscle-derived exosomes can influence insulin secretion deserves attention.

### Metabolites

The interaction between muscle and pancreas may not be restricted to myokines and exosomes. Metabolites such as lactate may also participate, considering that skeletal muscle plays a predominant role in lactate metabolism. In addition, we (Galgani et al., 2013) and others (Lovejoy et al., 1992) have found a direct correlation between plasma lactate concentration and insulin resistance. Thus, lactate might represent a candidate for linking skeletal muscle insulin sensitivity with insulin secretion. However, its actual relevance in driving insulin secretion is controversial. On the one hand, lactate showed to stimulate *in vitro* insulin secretion (Meats et al., 1989; Akiyoshi et al., 1999). On the other hand, lactate was reported to have a null effect on insulin secretion (Ishihara et al., 1999). The latter explained by lacking expression of the monocarboxylate transporter 1 in pancreatic beta cells (Ishihara et al., 1999; Schuit et al., 2012).

## Conclusion

For many years, the inverse relationship between insulin sensitivity and its secretion is known. However, an underlying mechanism relating these processes remains elusive. Strikingly, *in vivo* animal (Handschin et al., 2007; Hirner et al., 2008) and human (Mari et al., 2011; Halperin et al., 2012) studies show that changes in skeletal muscle glucose metabolism are accompanied by differential insulin secretion. Some of the evidence has been mostly interpreted as a direct role of insulin on its own secretion (Mari et al., 2011; Halperin et al., 2012). Because insulin drastically increases skeletal muscle glucose metabolism, we propose that a putative interaction between skeletal muscle and the pancreas may proceed. Assessing a causal relationship between these processes is challenging. It requires altering muscle glucose metabolism while maintaining similar blood glucose and insulin concentration. Under that setting, it will be essential to combine blood sampling from arterio-venous balance with proteomic analysis. Such assessment will contribute to identifying such an endocrine factor (or refute its existence) in conditions of physiological (e.g., transition from fasting to postprandial) and pathophysiological (e.g., normal vs. abnormal glycemic control) relevance.

The definition of myokine also needs to be revisited. At present, many muscle cell-derived proteins are named myokines. However, there is no evidence that those factors can indeed affect the function of any organ *in vivo*. Perhaps, the strongest evidence for supporting an endocrine role of a myokine on a distant organ is attributed to IL-6. Thus, in individuals undergoing a 12 week exercise training, blocking IL-6 action (by infusing an IL-6 receptor antibody) prevented the decrease in visceral fat mass as detected in participants receiving vehicle infusion (Wedell-Neergaard et al., 2018). Authors interpreted that finding as proof of the lipolytic action of IL-6 shown in earlier studies (van Hall et al., 2003). Certainly, Wedell-Neergaard’s study cannot claim that muscle-derived IL-6 was responsible of decreasing visceral fat mass. In any case, it is the strongest current evidence in humans linking skeletal muscle contraction with changes in a distant tissue throughout a circulating factor. Whether or not blocking IL-6 action had any effect on insulin secretion after exercise training deserves analysis.

Finally, one should bear in mind that besides skeletal muscle, organs such as the liver and adipose tissue, may also secrete a number of factors into circulation. Those factors may play a role in mediating the interaction between insulin sensitivity and its secretion. Identifying the mechanism linking insulin sensitivity with insulin secretion, in particular in dynamic states such as the transition from fasting to postprandial conditions, may have a strong impact on designing therapies for improving insulin secretion in pre-diabetic and diabetic individuals.

## Author Contributions

MM and JG conceived the present idea and drafted the manuscript. All authors revised and approved the final version of the manuscript.

## Conflict of Interest Statement

The authors declare that the research was conducted in the absence of any commercial or financial relationships that could be construed as a potential conflict of interest.
