# Supplementary material for: Insights on the Role of Putative Muscle-Derived Factors on Pancreatic Beta Cell Function
Source: Front Physiol. 2019 Aug 8;10:1024. doi: 10.3389/fphys.2019.01024 (PMC6694406; doi:10.3389/fphys.2019.01024)
Supplement: Supplementary file 1 [file Table_1.DOCX]

**Supplementary material**

**Suppl Table 1. Influence of manipulations of insulin signaling on *in vitro* insulin secretion.**

| **Study** | **Model** | **Specie** | **Experimental design** | | | | **Finding**  **(vs. control)** | **Interpretation of the effect of insulin on its secretion** |
| --- | --- | --- | --- | --- | --- | --- | --- | --- |
|  |  |  | **Treatment** | **Glucose, mM** | **Time, min** | **Assessment** |  |  |
| (1) | Beta cells | Mouse  Canine  Human  Porcine | Insulin 0.1 µM | 3 | <0.5 | e.c. [serotonin] | ↑ | Stimulates |
| (2) | Islets | Mouse | Wortmannin 0.1 µM | 16.7 | 60 | e.c. [insulin] | ↑ | Inhibits |
| (3) | Beta cells | Mouse | Insulin 0.001 - 0.6 µM | 10 | 7 | Electrical activity  Membrane potential | ↓ | Inhibits |
| (3) | In situ beta cells* | Mouse | Insulin 0.2 µM | 10 | 7 | Electrical activity  Membrane potential | ↓ | Inhibits |
| (3) | In situ beta cells* | Mouse | Insulin 0.1 µM | 10 | 9 | i.c. [Ca^2+^] | ↓ | Inhibits |
| (4) | Islets | Rat | Insulin 0.1 µM | 8 | 40 | e.c. [C-peptide] | - | None |
| (4) | Islets | Rat | Insulin 0.1 - 0.5 µM | 3 | 30 | e.c. [C-peptide] | - | None |
| (5) | Islets | Human | Insulin-mimetic 10 µM | 2 | 60 | e.c. [insulin] | ↓ | Inhibits |
| (6) | Perfused pancreas | Dog | Insulin 3 nM | 8.3 | 20 | e.c. [insulin] | ↓ | Inhibits |
| (7) | Islets | Rat | IRS1 antisense oligonucleotide 4 nM | 11 | 30-60 | e.c. [insulin] | ↑ | Inhibits |
| (7) | Islets | Rat | IRS1 antisense oligonucleotide 4 nM | 16.7 | 30-60 | e.c. [insulin] | ↑ | Inhibits |
| (8) | INS1 cells | Rat | PI3K inhibitor (LY294002 50 µM) | 16 | Not specify | e.c. [insulin] | ↑ | Inhibits |
| (8) | INS1 cells | Rat | PI3K inhibitor (Wortmannin 0.05 µM) | 16 | Not specify | e.c. [insulin] | - | None |
| (9) | Islets | Mouse | Anti-insulin 1 µg/mL | 25 | 30 | e.c. [insulin] | ↓ | Stimulates |
| (10) | INS1E cells | Rat | Insulin receptor inhibitor (ENPP-1) | 16.7 | 60 | e.c. [insulin] | ↓ | Stimulates |
| (11) | Islet | Mouse | Insulin 0.05 - 0.1 nM | 8.3 | 60 | e.c. [C-peptide] | ↑ | Stimulates |
| (11) | Islet | Mouse | Insulin 1 µM | 8.3 | 60 | e.c. [C-peptide] | ↓ | Inhibits |
| (12) | Islet | Human | Insulin 2 µM | 15 | 30 | e.c. [C-peptide] | ↓ | Inhibits |

Conversion factor used for insulin concentration: 1 µIU/mL = 6.945 pmol/L

e.c./i.c. [nn] extra-, intra-cellular concentration

* In situ beta cells: beta cells within whole islet

**Table S1 References**

1. Aspinwall CA, Lakey JR, Kennedy RT. Insulin-stimulated insulin secretion in single pancreatic beta cells. J Biol Chem 1999;274:6360-6365

2. Mizgier ML, Gutierrez J , Galgani JE. Unpublished

3. Khan FA, Goforth PB, Zhang M, Satin LS. Insulin activates ATP-sensitive K(+) channels in pancreatic beta-cells through a phosphatidylinositol 3-kinase-dependent pathway. Diabetes 2001;50:2192-2198

4. Zawalich WS, Zawalich KC. Effects of glucose, exogenous insulin, and carbachol on C-peptide and insulin secretion from isolated perifused rat islets. J Biol Chem 2002;277:26233-26237

5. Persaud SJ, Asare-Anane H, Jones PM. Insulin receptor activation inhibits insulin secretion from human islets of Langerhans. FEBS Lett 2002;510:225-228

6. Iversen J, Miles DW: Evidence for a feedback inhibition of insulin on insulin secretion in the isolated, perfused canine pancreas. Diabetes 1971;20:1-9

7. [Araujo EP](https://www.ncbi.nlm.nih.gov/pubmed/?term=Araujo%20EP%5BAuthor%5D&cauthor=true&cauthor_uid=12435589), [Amaral ME](https://www.ncbi.nlm.nih.gov/pubmed/?term=Amaral%20ME%5BAuthor%5D&cauthor=true&cauthor_uid=12435589), [Souza CT](https://www.ncbi.nlm.nih.gov/pubmed/?term=Souza%20CT%5BAuthor%5D&cauthor=true&cauthor_uid=12435589), [Bordin S](https://www.ncbi.nlm.nih.gov/pubmed/?term=Bordin%20S%5BAuthor%5D&cauthor=true&cauthor_uid=12435589), [Ferreira F](https://www.ncbi.nlm.nih.gov/pubmed/?term=Ferreira%20F%5BAuthor%5D&cauthor=true&cauthor_uid=12435589), [Saad MJ](https://www.ncbi.nlm.nih.gov/pubmed/?term=Saad%20MJ%5BAuthor%5D&cauthor=true&cauthor_uid=12435589), [Boschero AC](https://www.ncbi.nlm.nih.gov/pubmed/?term=Boschero%20AC%5BAuthor%5D&cauthor=true&cauthor_uid=12435589), [Magalhães EC](https://www.ncbi.nlm.nih.gov/pubmed/?term=Magalh%C3%A3es%20EC%5BAuthor%5D&cauthor=true&cauthor_uid=12435589), [Velloso LA](https://www.ncbi.nlm.nih.gov/pubmed/?term=Velloso%20LA%5BAuthor%5D&cauthor=true&cauthor_uid=12435589): Blockade of IRS1 in isolated rat pancreatic islets improves glucose-induced insulin secretion. [FEBS Lett.](https://www.ncbi.nlm.nih.gov/pubmed/12435589) 2002 Nov 20;531(3):437-42.

8. [Collier JJ](https://www.ncbi.nlm.nih.gov/pubmed/?term=Collier%20JJ%5BAuthor%5D&cauthor=true&cauthor_uid=15485656), [White SM](https://www.ncbi.nlm.nih.gov/pubmed/?term=White%20SM%5BAuthor%5D&cauthor=true&cauthor_uid=15485656), [Dick GM](https://www.ncbi.nlm.nih.gov/pubmed/?term=Dick%20GM%5BAuthor%5D&cauthor=true&cauthor_uid=15485656), [Scott DK](https://www.ncbi.nlm.nih.gov/pubmed/?term=Scott%20DK%5BAuthor%5D&cauthor=true&cauthor_uid=15485656). Phosphatidylinositol 3-kinase inhibitors reveal a unique mechanism of enhancing insulin secretion in 832/13 rat insulinoma cells. [Biochem Biophys Res Commun.](https://www.ncbi.nlm.nih.gov/pubmed/15485656) 2004 Nov 19;324(3):1018-23.

9. [Srivastava S](https://www.ncbi.nlm.nih.gov/pubmed/?term=Srivastava%20S%5BAuthor%5D&cauthor=true&cauthor_uid=12882922), [Goren HJ](https://www.ncbi.nlm.nih.gov/pubmed/?term=Goren%20HJ%5BAuthor%5D&cauthor=true&cauthor_uid=12882922). Insulin constitutively secreted by beta-cells is necessary for glucose-stimulated insulin secretion. [Diabetes.](https://www.ncbi.nlm.nih.gov/pubmed/?term=srivastava+s%2C+goren+hj+2003) 2003 Aug;52(8):2049-56.

10. Caporarello N, Frittitta L. Insulin receptor signaling and glucagon-like peptide 1 effects on pancreatic beta cells. PLoS One. 2017 Aug 2;12(8):e0181190

PLOS

11. Jimenez-Feltstrom J, Salehi A. Insulin feedback actions: complex effects involving isoforms of islet nitric oxide synthase. Regul Pept. 2004 Oct 15;122(2):109-18.

12. Johnson JD, Misler S. Nicotinic acid-adenine dinucleotide phosphate-sensitive calcium stores initiate insulin signaling in human beta cells. Proc Natl Acad Sci U S A. 2002 Oct 29;99(22):14566-71. Epub 2002 Oct 15.

**Suppl Table 2. Comparison of the presence of chemokines and cytokines in human myotube conditioned media by using different analytical approaches.**

| **Protein** | **Gene** | **Uniprot identifier** | **Mizgier et al., 2017** | | | **Scheler et al., 2013** | **Hartwig et al., 2014** | | | |
| --- | --- | --- | --- | --- | --- | --- | --- | --- | --- | --- |
|  |  |  | **Cut-off 1** | **Cut-off 2** | **MIA** | **MIA** | **MIA** | **LC-MS/MS** | **2D-MALDI-MS** | **LC-MS^E^** |
| CCL-23 | CCL23 | P55773 | ✔ | ✔ | NM | NM | - | - | - | - |
| ENA-78 | CXCL5 | P42830 | ✔ | ✔ | NM | NM | - | - | - | - |
| EOTAXIN-2 | CCL24 | O00175 | ✔ | ✔ | NM | NM | - | - | - | - |
| FGF-9 | FGF9 | P31371 | ✔ | ✔ | NM | NM | - | - | - | - |
| GCP-2 | CXCL6 | P80162 | ✔ | ✔ | NM | NM | - | - | - | - |
| GDNF | GDNF | P39905 | ✔ | ✔ | NM | NM | - | - | - | - |
| GRO-α/β/γ | CXCL1/2/3 | P09341/P19875/P19876 | ✔ | ✔ | NM | ✔ | ✔ | - | - | - |
| HGF | HGF | P14210 | ✔ | ✔ | NM | NM | - | - | - | - |
| IL-10 | IL10 | P22301 | ✔ | ✔ | NM | ✔ | ✔ | - | - | - |
| IL-16 | IL16 | Q14005 | ✔ | ✔ | NM | NM | - | - | - | - |
| IL-1α | IL1A | P01583 | ✔ | ✔ | NM | NM | - | - | - | - |
| IL-1β | IL1B | P01584 | ✔ | ✔ | NM | ✔ | ✔ | - | - | - |
| IL-3 | IL3 | P08700 | ✔ | ✔ | NM | NM | - | - | - | - |
| IL-6 | IL6 | P05231 | ✔ | ✔ | ✔ | ✔ | ✔ | - | - | - |
| IL-8 | CXCL8 | P10145 | ✔ | ✔ | ✔ | ✔ | ✔ | - | - | - |
| IP-10 | CXCL10 | P02778 | ✔ | ✔ | NM | NM | - | - | - | - |
| LIF | LIF | P15018 | ✔ | ✔ | NM | ✔ | ✔ | - | - | - |
| MCP-1 | CCL2 | P13500 | ✔ | ✔ | ✔ | ✔ | ✔ | - | - | - |
| M-CSF | CSF1 | P09603 | ✔ | ✔ | NM | NM | - | ✔ | - | ✔ |
| MIF | MIF | P14174 | ✔ | ✔ | ✔ | NM | - | ✔ | - | ✔ |
| MIP-1 | CCL4 | P13236 | ✔ | ✔ | NM | - | - | - | - | - |
| MIP-3α | CCL20 | P78556 | ✔ | ✔ | NM | NM | - | - | - | - |
| NAP-2 | PPBP | P02775 | ✔ | ✔ | NM | NM | - | - | - | - |
| NT-3 | NTF3 | P20783 | ✔ | ✔ | NM | NM | - | - | - | - |
| OSM | OSM | P13725 | ✔ | ✔ | NM | NM | - | - | - | - |
| PLGF | PGF | P49763 | ✔ | ✔ | NM | NM | - | - | - | - |
| RANTES | CCL5 | P13501 | ✔ | ✔ | ✔ | NM | - | - | - | - |
| TGFβ-2 | TGFB2 | P61812 | ✔ | ✔ | NM | NM | - | - | - | - |
| IGFBP-1 | IGFBP1 | [P08833](https://www.uniprot.org/uniprot/P08833) | ✔ | ✔ | NM | NM | - | - | - | - |
| IGFBP-2 | IGFBP2 | [P18065](https://www.uniprot.org/uniprot/P18065) | ✔ | ✔ | NM | NM | ✔ | - | ✔ | - |
| TIMP-1 | TIMP1 | [P01033](https://www.uniprot.org/uniprot/P01033) | ✔ | ✔ | NM | NM | ✔ | ✔ | ✔ | - |
| TIMP-2 | TIMP2 | [P16035](https://www.uniprot.org/uniprot/P16035) | ✔ | ✔ | NM | NM | ✔ | - | ✔ | - |
| ANG | ANG | P03950 | ✔ | - | NM | NM | - | - | - | - |
| BDNF | BDNF | P23560 | ✔ | - | NM | NM | - | - | - | - |
| BLC | CXCL13 | O43927 | ✔ | - | NM | NM | - | - | - | - |
| EOTAXIN-1 | CCL11 | P51671 | ✔ | - | NM | NM | - | - | - | - |
| GM-CSF | CSF2 | P04141 | ✔ | - | NM | - | - | - | - | - |
| I-309 | CCL1 | P22362 | ✔ | - | NM | NM | - | - | - | - |
| IFNγ | IFNG | P01579 | ✔ | - | - | ✔ | ✔ | - | - | - |
| IL-12 | IL12A/B | P29459/P29460 | ✔ | - | - | ✔ | ✔ | - | - | - |
| IL-15 | IL15 | P40933 | ✔ | - | NM | - | - | - | - | - |
| IL-2 | IL2 | P60568 | ✔ | - | NM | - | ✔ | - | - | - |
| IL-7 | IL7 | P13232 | ✔ | - | NM | ✔ | ✔ | - | - | - |
| LIGHT | TNFSF14 | O43557 | ✔ | - | NM | NM | - | - | - | - |
| MCP-2 | CCL8 | P80075 | ✔ | - | NM | NM | - | - | - | - |
| MCP-4 | CCL13 | Q99616 | ✔ | - | NM | NM | - | - | - | - |
| MDC | CCL22 | O00626 | ✔ | - | NM | NM | - | - | - | - |
| OPG | TNFRSF11B | O00300 | ✔ | - | NM | NM | - | - | - | - |
| OPN | SPP1 | P10451 | ✔ | - | NM | NM | - | - | - | - |
| PARC | CCL18 | P55774 | ✔ | - | NM | NM | - | - | - | - |
| PDGF BB | PDGFB | P01127 | ✔ | - | NM | NM | - | - | - | - |
| SCF | KITLG | P21583 | ✔ | - | NM | NM | - | - | - | - |
| SDF-1 | CXCL12 | P48061 | ✔ | - | NM | NM | - | - | - | - |
| TARC | CCL17 | Q92583 | ✔ | - | NM | NM | - | - | - | - |
| TGFβ-3 | TGFB3 | P10600 | ✔ | - | NM | NM | - | - | - | - |
| TNF-β | LTA | P01374 | ✔ | - | NM | NM | - | - | - | - |
| VEGF | VEGFA | P15692 | ✔ | - | NM | ✔ | ✔ | - | - | - |
| EGF | EGF | P01133 | - | - | NM | NM | - | - | - | - |
| EOTAXIN-3 | CCL26 | Q9Y258 | - | - | NM | NM | - | - | - | - |
| FGF-4 | FGF4 | P08620 | - | - | NM | NM | - | - | - | - |
| FGF-6 | FGF6 | P10767 | - | - | NM | NM | - | - | - | - |
| FGF-7 | FGF7 | P21781 | - | - | NM | NM | - | - | - | - |
| Flt3 Ligand | Flt3lg | P49772 | - | - | NM | NM | - | - | - | - |
| Fractalkine | CX3CL1 | P78423 | - | - | - | NM | - | - | - | - |
| G-CSF | CSF3 | P09919 | - | - | NM | ✔ | ✔ | - | - | - |
| GRO-α | CXCL1 | P09341 | - | - | NM | NM | ✔ | - | - | - |
| IGF-1 | IGF1 | P05019 | - | - | NM | NM | - | - | - | - |
| IL-13 | IL13 | P35225 | - | - | NM | ✔ | ✔ | - | - | - |
| IL-4 | IL4 | P05112 | - | - | NM | ✔ | ✔ | - | - | - |
| IL-5 | IL5 | P05113 | - | - | NM | ✔ | - | - | - | - |
| MCP-3 | CCL7 | P80098 | - | - | NM | NM | - | - | - | - |
| MIG | CXCL9 | Q07325 | - | - | NM | NM | - | - | - | - |
| MIP-1δ | CCL15 | Q16663 | - | - | NM | NM | - | - | - | - |
| NT-4 | NTF4 | P34130 | - | - | NM | NM | - | - | - | - |
| TGFβ-1 | TGFB1 | P01137 | - | - | NM | NM | - | - | - | - |
| THPO | THPO | P40225 | - | - | NM | NM | - | - | - | - |
| TNF-α | TNF | P01375 | - | - | NM | ✔ | ✔ | - | - | - |
| IGFBP-3 | IGFBP3 | [P17936](https://www.uniprot.org/uniprot/P17936) | - | - | NM | NM | - | - | ✔ | - |
| IGFBP-4 | IGFBP4 | [P22692](https://www.uniprot.org/uniprot/P22692) | - | - | NM | NM | ✔ | - | ✔ | - |
| LEPTIN | LEP | [P41159](https://www.uniprot.org/uniprot/P41159) | - | - | NM | NM | - | - | - | - |

**Legend for Suppl. Table 2**

Protein analysis by using a commercial array (Human cytokine array C5, Raybiotech) and multiplex immunoassay ([MIA], Luminex, R&D System) (Mizgier et al., 2017). Scheler et al. (Scheler et al., 2013) conducted MIA (Bio-plex 200, Biorad). Hartwig et al. (Hartwig et al., 2014) conducted MIA (Bio-Plex Pro, Biorad) and non-targeted proteomic analyses through i) liquid chromatography with tandem mass spectroscopy (LC/MS/MS); ii) two-dimensional gel electrophoresis matrix assisted laser desorption/ionization mass spectrometry (2D-MALDI/MS); and iii) data independent liquid chromatography mass spectrometry MS^E^ engine (LC/MS^E^). *Cut-off point 1 and 2 were defined as 2- and 3-fold above negative control, respectively. Detected (✔); Not detected (-); Not measured (NM).

**Table S2 References**

Hartwig, S., Raschke, S., Knebel, B., Scheler, M., Irmler, M., Passlack, W., et al. (2014). Secretome profiling of primary human skeletal muscle cells. Biochim Biophys Acta 1844(5), 1011-1017. doi: 10.1016/j.bbapap.2013.08.004.

Mizgier, M.L., Cataldo, L.R., Gutierrez, J., Santos, J.L., Casas, M., Llanos, P., et al. (2017). Effect of human myotubes-derived media on glucose-stimulated insulin secretion. Journal of Diabetes Research In press.

Scheler, M., Irmler, M., Lehr, S., Hartwig, S., Staiger, H., Al-Hasani, H., et al. (2013). Cytokine response of primary human myotubes in an in vitro exercise model. Am J Physiol Cell Physiol 305(8), C877-886. doi: 10.1152/ajpcell.00043.2013.

**Suppl. Table 3. Function and existence of the receptor for human myotube-derived proteins.**

| **Protein** | **Gene** | **Uniprot identifier** | **Receptor** | **Function** |
| --- | --- | --- | --- | --- |
|  |  |  |  |  |
| Chloride channel 27 | CLIC1 | O00299 | Not reported | Ion channel |
| Sulfhydryloxidase 1 | QSOX1 | O00391 | Not reported | Oxidoreductase |
| Importin 5 | IPO5 | O00410 | Not reported | Protein transport |
| β -mannosidase | MANBA | O00462 | Not reported | Hydrolase |
| Myc Box interacting protein 1 | BIN1 | O00499 | Not reported | Nucleocytoplasmic adaptor |
| Syntenin 1 | SDCBP | O00560 | Not reported | Cytoskeleton |
| Pyridoxal kinase | PDXK | O00764 | Not reported | Kinase |
| Heat shock protein β6 | HSPB6 | O14558 | Not reported | Stress response |
| Coatomer subunit ε | COPE | O14579 | Not reported | Vesicle mediated transport |
| Tripeptidylpeptidase 1 | TPP1 | O14773 | Not reported | Hydrolase |
| Proteasome subunit α7 | PSMA7 | O14818 | Not reported | Proteasome |
| Myosin light chain 12B | MYL12B | O14950 | Not reported | Motor protein |
| ARP 2/3 subunit 2 | ARPC2 | O15144 | Not reported | Cytoskeleton |
| ARP 2/3 subunit 3 | ARPC3 | O15145 | Not reported | Cytoskeleton |
| Proteasome subunit p58 | PSMD3 | O43242 | Not reported | Proteasome |
| Thioredoxin-like protein 1 | TXNL1 | O43396 | Not reported | Oxidoreductase |
| Alpha-actinin 4 | ACTN4 | O43707 | Not reported | Structural |
| Calumenin | CALU | O43852 | Not reported | Calcium binding |
| NDP kinase | NME2P1 | O60361 | Not reported | Kinase |
| Sarcosin | KLHL41 | O60662 | Not reported | Skeletal muscle development |
| Perilipin 3 | PLIN3 | O60664 | Not reported | Vesicle mediated transport |
| SRPX2 | SRPX2 | O60687 | Not reported | Angiogenesis; Cell adhesion |
| Actin-interacting protein 1 | WDR1 | O75083 | Not reported | Cytoskeleton |
| γ-glutamylcyclotransferase | GGCT | O75223 | Not reported | Transferase |
| SH3 binding glutamic acid-rich-like protein | SH3BGRL | O75368 | Not reported | Oxidoreductase (putative) |
| Filamin B | FLNB | O75369 | Not reported | Cytoskeleton |
| Isocitrate dehydrogenase | IDH1 | O75874 | Not reported | Oxidoreductase |
| Stanniocalcin 2 | STC2 | O76061 | Not reported | Hormone (calcium homeostasis) |
| Dimethylarginine dimethylaminohydrolase 1 | DDAH1 | O94760 | Not reported | Hydrolase |
| Calsyntenin 1 | CLSTN1 | O94985 | Not reported | Structural/adhesion |
| 6-phosphogluconolactonase | PGLS | O95336 | Not reported | Hydrolase |
| Keratin, type II cytoskeletal 75 | KRT75 | O95678 | Not reported | Cytoskeleton |
| Bisphosphate 3'-nucleotidase 1 | BPNT1 | O95861 | Not reported | Hydrolase |
| Dimethylarginine dimethylaminohydrolase 2 | DDAH2 | O95865 | Not reported | Hydrolase |
| Integrin β-like protein 1 | ITGBL1 | O95965 | Not reported | Cell adhesion |
| Alcohol dehydrogenase 1B | ADH1B | P00325 | Not reported | Oxidoreductase |
| L-lactate dehydrogenase A chain | LDHA | P00338 | Not reported | Oxidoreductase |
| Retinal dehydrogenase 1 | ALDH1A1 | P00352 | Not reported | Oxidoreductase |
| Glutathione reductase (mitochondrial) | GSR | P00390 | Not reported | Oxidoreductase |
| Superoxide dismutase | SOD1 | P00441 | Not reported | Oxidoreductase |
| Coagulation factor XIII A chain | F13A1 | P00488 | Not reported | Transferase |
| Aspartate aminotransferase mitochondrial | GOT2 | P00505 | Not reported | Transferase |
| Phosphoglycerate kinase 1 | PGK1 | P00558 | Not reported | Kinase |
| Adenylate kinase isoenzyme 1 | AK1 | P00568 | Not reported | Kinase |
| Complement C1r subcomponent | C1R | P00736 | Not reported | Hydrolase |
| Complement factor D | CFD | P00746 | Not reported | Hydrolase |
| Complement factor B | CFB | P00751 | Not reported | Hydrolase |
| Adenosine deaminase | ADA | P00813 | Not reported | Hydrolase |
| Antithrombin-III | SERPINC1 | P01008 | Not reported | Protease inhibitor |
| Alpha-2-macroglobulin | A2M | P01023 | Not reported | Protease inhibitor |
| Complement C3 | C3 | P01024 | CR1, CR2, C3AR | Complement pathway |
| Metalloproteinase inhibitor 1 | TIMP1 | P01033 | Not reported | Protease inhibitor |
| Cystatin C | CST3 | P01034 | Not reported | Protease inhibitor |
| Tumor necrosis factor | TNF | P01375 | TNFRSF1A, TNFSF1B | Cytokine |
| Interferon γ | IFNG | P01579 | INFGR1, IFNGR2 | Cytokine |
| Interleukin 1 β | IL1B | P01584 | IL1R1, IL1R2 | Cytokine |
| Collagen α-1(I) chain | COL1A1 | P02452 | Not reported | Extracellular matrix structure |
| Collagen α-1(III) chain | COL3A1 | P02461 | Not reported | Extracellular matrix structure |
| α-crystallin B chain | CRYAB | P02511 | Not reported | Chaperone |
| Keratin, type I cytoskeletal 14 | KRT14 | P02533 | Not reported | Cytoskeleton |
| Keratin, type II cytoskeletal 6A | KRT6A | P02538 | Not reported | Cytoskeleton |

**Legend for Suppl. Table 3**

From the first 60 human myotube-derived proteins detected by Hartwig et al. ([Hartwig et al., 2014](#_ENREF_1)), the existence of their receptor was searched using UniProt and STRING (score >0.95 for binding and activation) databases. Function was identified using UniProt database.
